# Dynamics of Choline-Containing Phospholipids in Traumatic Brain Injury and Associated Comorbidities

**DOI:** 10.3390/ijms222111313

**Published:** 2021-10-20

**Authors:** Sana Javaid, Talha Farooq, Zohabia Rehman, Ammara Afzal, Waseem Ashraf, Muhammad Fawad Rasool, Faleh Alqahtani, Sary Alsanea, Fawaz Alasmari, Mohammed Mufadhe Alanazi, Metab Alharbi, Imran Imran

**Affiliations:** 1 Department of Pharmacology, Faculty of Pharmacy, Bahauddin Zakariya University, Multan 60800, Pakistan; sana.javaid@wum.edu.pk (S.J.); talha.cr999@gmail.com (T.F.); zoharehman818@gmail.com (Z.R.); ammara.afzal60@gmail.com (A.A.); chishtiwaseem@yahoo.com (W.A.); Imran.ch@bzu.edu.pk (I.I.); 2 Department of Pharmacy, The Women University, Multan 60000, Pakistan; 3 Department of Pharmacy Practice, Faculty of Pharmacy, Bahauddin Zakariya University, Multan 60800, Pakistan; fawadrasool@bzu.edu.pk; 4 Department of Pharmacology and Toxicology, College of Pharmacy, King Saud University, Riyadh 11451, Saudi Arabia; Salsanea@ksu.edu.sa (S.A.); ffalasmari@ksu.edu.sa (F.A.); momalanazi@ksu.edu.sa (M.M.A.); mesalharbi@ksu.edu.sa (M.A.)

**Keywords:** traumatic brain injury, choline, phosphatidylcholine, brain phospholipids, citicholine, choline-targeted therapy

## Abstract

The incidences of traumatic brain injuries (TBIs) are increasing globally because of expanding population and increased dependencies on motorized vehicles and machines. This has resulted in increased socio-economic burden on the healthcare system, as TBIs are often associated with mental and physical morbidities with lifelong dependencies, and have severely limited therapeutic options. There is an emerging need to identify the molecular mechanisms orchestrating these injuries to life-long neurodegenerative disease and a therapeutic strategy to counter them. This review highlights the dynamics and role of choline-containing phospholipids during TBIs and how they can be used to evaluate the severity of injuries and later targeted to mitigate neuro-degradation, based on clinical and preclinical studies. Choline-based phospholipids are involved in maintaining the structural integrity of the neuronal/glial cell membranes and are simultaneously the essential component of various biochemical pathways, such as cholinergic neuronal transmission in the brain. Choline or its metabolite levels increase during acute and chronic phases of TBI because of excitotoxicity, ischemia and oxidative stress; this can serve as useful biomarker to predict the severity and prognosis of TBIs. Moreover, the effect of choline-replenishing agents as a post-TBI management strategy has been reviewed in clinical and preclinical studies. Overall, this review determines the theranostic potential of choline phospholipids and provides new insights in the management of TBI.

## 1. Introduction

Traumatic brain injury (TBI) is the physiological disruption of the central nervous system due to a sudden blow to the brain resulting in physical and neurological incapacity, sometimes leading to life-long disability and death. The leading causes include accidental falls, sports injuries and vehicle collisions [1]. This silent epidemic affects people of all age groups and is reported to victimize sixty-nine million of the world’s populace annually [2]. The prevailing incidence of TBI imposes the burden of morbidity and mortality on the insufficiently prepared health system of developing countries. Although the health care system and research in the medical field are improving over time, the exact prediction of TBI-imposed damages on the entire global health system is still challenging [3]. The experts find brain injuries very difficult to manage due to availability of limited therapeutic options. Diuretics are used to reduce the post-TBI accumulation of fluid in brain while corticosteroids halt the progression of secondary injuries by inhibiting the PLA2/COX/LOX pathways [4].

**Figure 1:**
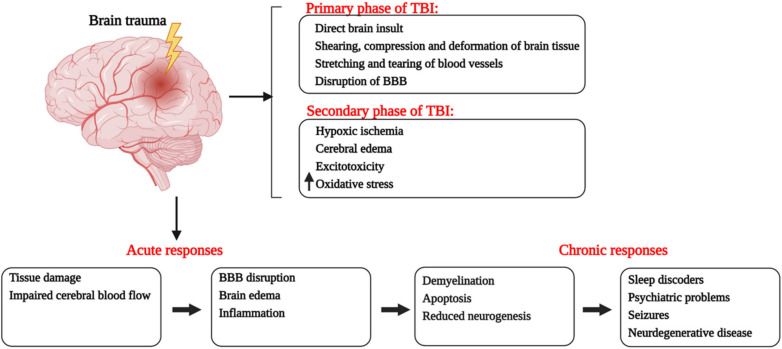
Pathological events happening during primary and secondary phases of traumatic brain injury with a description of short-term and long-term consequences of brain trauma. Red font is showing the phases of TBI.↑ shows the increased oxidative stress. The figure was created with BioRender.com (accessed on 9 September 2021).

There are two distinct phases of TBI-induced brain damage, categorized as primary and secondary [5], summarized in Figure 1. The stage of primary injury involves the consequences of direct mechanical insult i.e., laceration, skull fractures, contusion of cerebral tissues and neuronal compression leading to subarachnoid or intracranial hemorrhage [6]. After hours or days, these primary offenses cause the initiation of a series of processes that contribute towards damage to the blood-brain barrier and loss of cerebral autoregulation [7]. Hypotension and ischemia hypoxia are prominent factors that cause disrupted blood flow, impaired oxygenation and eventual death of brain tissue [8]. The increased intracranial pressure due to cerebral edema causes brainstem compression and diffused brain injury [9]. Additionally, the second phase also includes the imbalanced neurotransmission and over-activation of biochemical receptors that result in excitotoxicity and neurodegeneration. TBI induces excessive release of glutamate and aspartate from presynaptic neurons as well as the reduced uptake of glutamate, due to the declined expression of glutamate transporters [10]. These changes result in the hyperactivation of NMDA (N-methyl-D-aspartate) and AMPA (α-Amino-3-hydroxy-5-methyl-4-isoxazolepropionic acid) receptors and modify the ion homeostasis in postsynaptic nerve endings. Increased intracellular Ca^2+^ results further cause the activation of various enzymes, resulting in neuronal death [11]. Additionally, the activation of NMDA receptors also causes the production of reactive oxidative species collectively leading to mitochondrial dysfunction [12].

On the basis of severity of the injury, TBI can also be classified as mild, moderate and severe by employing the Glasgow Coma Scale (GCS). The GCS comprises the examination of patients in the acute phase of injury to inspect the opening of eye, vocal and motor responses for assessment of patient’s consciousness [13]. Post-traumatic amnesia (PTA) is another way to judge the severity of TBI, which involves the estimation of the patient’s state of confusion after recovery from unconsciousness. The PTA of less than 24 h is categorized as mild, falling between 24 h to one week as moderate and extending beyond is classified as severe TBI (Table 1 and Table 2) [14].

## 2. Neurobiological Significance of Lipids

Lipids are essential for the structural and functional integrity of the central nervous system and account for up to 45% of the dry weight of the brain [15]. The brain has the highest content of lipids after adipose tissue [16]. These macromolecules are directly involved in brain homeostasis and various neuronal processes due to their role in synaptogenesis, neurogenesis, impulse and signal transduction. In the brain, the lipids are majorly categorized as cholesterol, glycerophospholipids and sphingolipids [17].

The brain is rich in sphingolipids, which are crucial for the development and function integrity of the CNS. The brain composition of sphingolipids continues to fluctuate as the brain develops and ages [18]. The subclass gangliosides are abundant in grey matter and neurons while sphingomyelin (SM), galactosyl-ceramide and sulfatide are rich in myelin sheath and oligodendrocytes [19].

**Figure 2:**
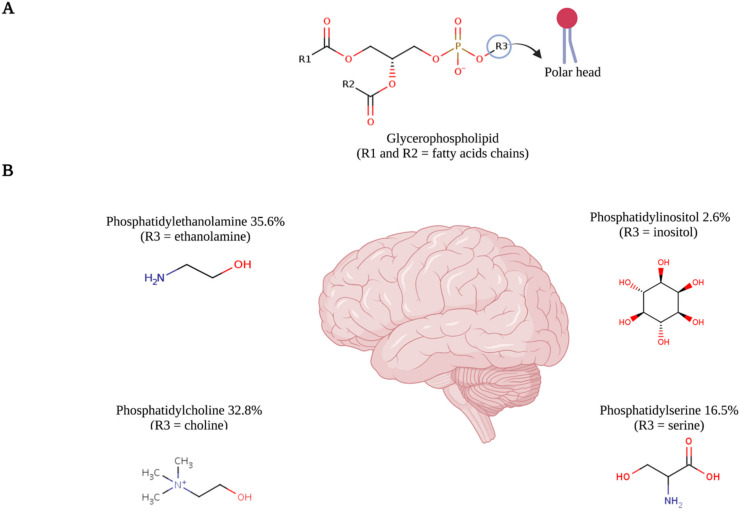
The illustration of (**A**) molecular structure of glycerophospholipid, comprising a glycerol molecule esterified with two fatty acids (R1 and R2), i.e., arachidonic acid and docosahexaenoic acid. One phosphate group and (**B**) structural details of R3 group yield different subtypes of glycerophospholipids with their % content of total glycerophospholipids in the brain [20]. Red font is indicating the functional groups. This figure was created with BioRender.com (accessed on 9 September 2021) and chemical structures were adapted from https://www.ebi.ac.uk (accessed on 9 September 2021).

Glycerophospholipids, also known as phosphoglycerides, are fatty acid diglycerides with a phosphatidyl ester attached to the terminal carbon. Approximately 4–5% of the total wet weight of the brain, including 4.2% of grey matter and 7% of white matter, is represented by glycerophospholipids categorized as phosphatidylcholine, phosphatidylethanolamine, phosphatidylglycerol and phosphatidylserine [20] (Figure 2). The structural diversity of these glycerophospholipids plays a pivotal role in the fluidity and stability of neuronal membranes which if disturbed, might result in neurological trouble. These glycerophospholipids also act as the reservoirs of secondary messengers as their breakdown by phospholipases results in the production of eicosanoids, prostaglandins, diacylglycerol and platelet-activating factors. They are also involved in apoptosis, modulation of activities of transporters and membrane-bound enzymes [21].

Sphingolipids are the lipids comprising sphingoid-base backbone; its sub-types are sphingomyelins, ceramides, and glycosphingolipids [15]. In the brain, sphingolipids are a vital component of the neuronal membrane as well as essential for neurogenesis, synaptogenesis, synaptic transmission and myelin stability. The altered metabolism of sphingolipids resulting from their disturbed degradation or biosynthesis is reported to be involved in many neurological disorders [22]. In the outer layer of neuronal cell membranes, phosphatidylcholine and sphingomyelin are in excess while the inner layer is rich in phosphatidylserine, phosphatidylinositol and phosphatidylethanolamine.

The brain is enriched with two polyunsaturated fatty acids (PUFs) named docosahexaenoic acid and arachidonic acid. These PUFs are found esterified with phospholipids of the cell membrane and get released after the neuroreceptor activation to take part in signal transduction. These two PUFs play a vital role in neurotransmission, neuroinflammation, neuronal survival and normal synaptic functionality [23].

## 3. TBI-Induced Pathophysiological Changes in Brain Phospholipids

The brain comprises lipids as its chief component and 44% of myelin is composed of phospholipids. Thus, the role of phospholipases in brain trauma is substantial, as these enzymes act as a convergent molecule for multiple mechanisms involved in the pathogenesis of TBI. TBI-induced exaggerated action of phospholipase A_2_ (PLA_2_) activation causes the breakdown of membrane glycerophospholipids, resulting in the generation of free fatty acids and lysophospholipids [24]. This action of PLA_2_ plays a crucial role in the pathogenesis of TBI, as derived fatty acids act as a substrate for cyclooxygenases to produce eicosanoids, which further aggravate the neuroinflammation [25].

The other metabolite generated, i.e., lysophospholipid, is known to disturb the fluidity and penetrability of the membrane [26]. Furthermore, the liberated FFAs with their metabolic products play a damaging role in promoting oxidative stress, consequently resulting in exacerbation of the secondary injury process after TBI. Moreover, the additionally generated bioactive products, i.e., lysophosphatidylcholine (lyso-PC) and lysophosphatidic acid, are converted to platelet activation factors, another important mediator of neuronal injury [24]. Membrane breakdown also builds up the oxidative stress in traumatic brain injury with increased isoprostanes generation from arachidonic acid, which are known be one of the most reliable markers of oxidative stress [27].

**Figure 3:**
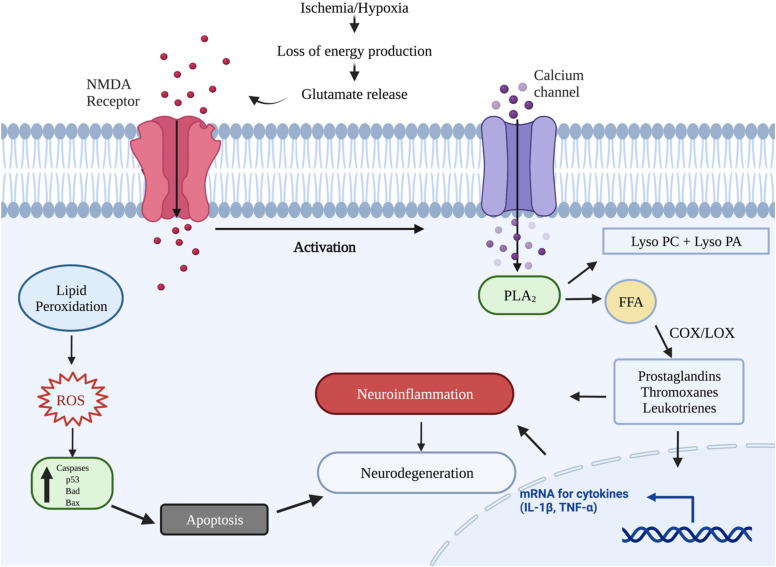
The underlying pathophysiological changes after TBI leading to neuroinflammation, increased oxidative stress and neuronal death. The increased oxygen requirements of the brain remain unmet due to TBI-induced hypoxia and ischemia that cause increased lipid peroxidation, which generate reactive oxygen species (ROS) and upregulation of pro-apoptotic proteins. The increased glutamate results in increased Ca^2+^ uptake and excitotoxicity, resulting in mitochondrial dysfunction and necrotic cell death. The overactivated phospholipase A_2_ causes the catalysis of membrane phospholipids into lysophosphatidylcholine (lyso-PC), lysophosphatidic acid (lyso PA) and free fatty acids i.e., arachidonic acid. These primary metabolites are bioactive and converted in platelet activating factors. The arachidonic acid undergoes the COX/LOX pathway to yield eicosanoids causing upregulation of inflammatory cytokines. Red dots are showing the Glutamate neurotransmitter and purple dots are showing the Calcium. This figure was created with BioRender.com (accessed on 9 September 2021).

Subsequent to traumatic injury, the brain has increased vulnerability of enzymatic [28] and non-enzymatic [29] lipid peroxidation due to its larger fatty acid content, increased oxygen requirements for appropriate metabolic activity and incapacity of the brain to regenerate [30]. Lipid peroxidation involves the insertion of a hydroperoxy group into PUFs constituents of phospholipids, causing damage to phospholipids which are crucial for intact cellular membranes [31]. There is sufficient evidence to reveal the parallel relationship between lipid peroxidation and the severity of traumatic brain injury. The deterioration of membrane integrity and permeability are the noticeable localized impact of lipid peroxidation [30]. Thus, exaggerated lipid peroxidation results in the accumulation of oxygenated fatty acids, leading to further damage. This oxidative degradation of membrane lipids can also initiate the secondary cellular responses, as these derived oxidized products are crucially associated with the disruption of the blood-brain barrier, dysregulation in cerebral blood flow, exaggeration of inflammatory reaction and neuronal apoptosis [32] (Figure 3).

## 4. Importance of Choline-Containing Phospholipids in Brain

Choline plays an essential role in the synthesis of different membrane phospholipids, i.e., phosphatidylcholine, choline plasmalogen and sphingomyelin. It also acts as a precursor for the synthesis of the neurotransmitter acetylcholine (Ach). Choline supplementation at each stage of brain development augments brain performance possibly because of membrane synthesis at the time of neuronal development, hence, it requires an adequate supply for adequate brain health. Its deficiency stimulates apoptosis and neuronal cell death and might precipitate atherosclerosis, neurological disorders and fatty liver disorder [33]. Choline critically partakes during various neurochemical pathways. Being the predecessor of Ach, its role in brain disorders due to impaired cholinergic neurotransmission is broadly documented. The compromised cholinergic neurotransmission can also precipitate learning and memory impairment [34].

**Figure 4:**
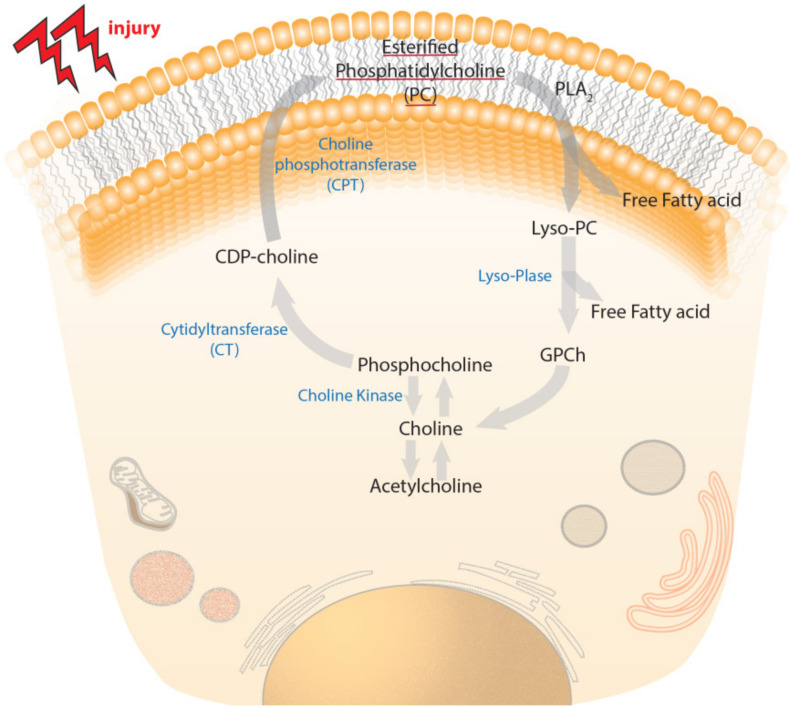
The depiction of biosynthesis and degradation of phosphatidylcholine. In the anabolic pathway, phosphorylation of choline takes place by choline kinase (CK), yielding phosphocholine, which is followed by condensation of phosphocholine catalyzed by cytidylyltransferase (CT), resulting in the formation of CDP-choline. Later, the coupling of phosphatidic acid and CDP-choline by choline-phosphotransferase (CPT) results in phosphatidylcholine synthesis. The breakdown of phosphatidylcholine results in the formation of lyso-phosphatidylcholine and free fatty acids (FFA) in the presence of PLA_2_. Lyso-PC quickly hydrolyzed to form FFA and glycerophosphocholine that form free choline, or phosphocholine through hydrolysis, by the action of alkaline phosphatase.

Phosphatidylcholine is the leading form of phosphoglycerides that comprises the choline molecule as the head group. It accounts for 32.8% of the total glycerophospholipid content of the human brain [20]. It is the major phospholipid present in the outer layer of the cellular and intracellular membranes of mammalian cells. The synthesis might be through direct methylation of the ethanolamine residue of phosphatidylethanolamine or via the Kennedy pathway. The choline is phosphorylated by choline kinases, which after processing by cytidylyltransferase, generated CDP-choline, which further couples with phosphatidic acid and gives phosphatidylcholine (Figure 4). The increased choline requirement during neuronal differentiation in order to synthesize new membrane is supported by several lines of evidence. Nowadays, it is clear that phosphatidylcholine and its metabolites play a signaling role during neuronal differentiation and might restore neuronal differentiation in many pathological conditions.

Other choline-containing PLs include sphingomyelin (SM) which is abundant in the myelin sheath and maintains the integrity of the axonal covering [35]. SM is also reported to take part in cellular processes, signal transduction and the inflammatory cascade [36]. SM also influences cognitive development through structural and functional contribution, as myelination is crucial for the maturation of brain networks and information processing [37]. Due to the acyl chain, sphingomyelin forms a cylindrical shape that is more narrow and tall than phosphatidylcholine, which results in the increased packing density of the membrane [38].

## 5. Changes in Choline-Containing Phospholipids after TBI

The brain injury resulting from trauma causes degradation of the cellular membrane that accounts for acute and chronic abnormalities. This pathological change causes the enzymatic breakdown of membrane phospholipids by activation of phospholipases [39]. The breakdown of phospholipids by the action of PLA_2_ yields glycero-PC and free fatty acids (Figure 4). These glycero-PC are bioactive and acetylated to produce a platelet activating factor, which further disrupts BBB, activates microglia and exacerbates neuroinflammation [40]. The TBI-induced hypoxia/ischemia also intensify the phospholipid and glycerol-PC breakdown, resulting in the release of choline during secondary injury mechanisms [33,41]. In addition to PLA_2_, the breakdown of phosphatidylcholine also takes place by the enzyme phospholipase D, which yields free choline and phosphatidic acid as breakdown products. Phosphatidic acid forms lysophosphatidic acid, which acts as a fibroblast growth factor. Phosphatidic acid also acts as a lipid second messenger and influences downstream enzymes, such as Raf kinase [42].

Metabolism of other choline-containing phospholipids also takes place in neural tissues, which is interconnected with the metabolism of phosphatidylcholine. The breakdown of sphingomyelin also results in the formation of phosphocholine and ceramide. Ceramide induces the process of apoptosis as a second messenger [43].

The elevated free choline levels in traumatized cortex and its surroundings are one of the most remarkable changes taking place during early TBI [44]. The TBI-mediated cerebral ischemia might increase the overall production of choline by either phospholipid catabolism by phospholipases or reduced clearance. The brain energy supply is also impaired after TBI. As phospholipid synthesis needs energy but degradation does not, this impairment in brain energy supply also increases the production of choline from phospholipids [45].

Post-TBI activation of phospholipases and the resulting variation in choline-phospholipids has been explored through numerous preclinical and clinical studies (Table 3). Homayoun et al. has reported the reduction in brain phospholipids at 4 and 35 days in rats after controlled cortical impact injury [46]. In the TBI model of controlled cortical impact damage, the lipidomic profile after 3 months of injury revealed the elevated phosphatidylcholine and sphingomyelin in the hippocampus, while these levels were decreased in the cerebellum and cortex of mice [47]. Ojo et al. examined the changes in different phospholipids and reported the elevation of phosphatidylcholine and sphingomyelin after mild-repetitive TBI in cortex and hippocampus during acute and chronic phases designated at time points of 24 h and 6–12 months, respectively [48]. The variation in plasma levels of phospholipids at different time points had also been analyzed in mouse models of closed head injuries, where decreased circulating phosphatidylcholine was recorded at 3 and 12 months of injury in comparison to their controls [49]. In another study by Scremin et al., the levels of choline were assessed after 24 h of cerebral cortex impact in rats. The outcomes revealed 700% of amplified choline levels at the injury site, suggesting that endogenous choline levels might be an early marker of TBI injury [45].

Pasvogel and their co-researcher attempted to provide clinical evidence for phosphatidylcholine variation in TBI. The outcomes of the study showed increased CSF levels of phosphatidylcholine in patients that were different from day 1 to 6 among alive and dead patients. The phosphatidylcholine was highest at 24 h after TBI and kept on decreasing in survivors till day 6. While its levels varied in persons who died and the levels were highest on the 4th day of TBI [50], these findings indicate that phospholipids breakdown is boosted in brains of patients deceased after brain trauma.

**Table 3:** Preclinical and clinical studies reporting the post-TBI changes in choline and choline-containing phospholipids.

**Preclinical Studies**				
**Animal**	**Brain Insult**	**Choline-Phospholipids Levels**	**Authors**	**Ref.**
SD rats	Controlled cortical impact injury	↓ PCh and GPC in the pericontusional zone at 2 and 4 h after injury	Xu et al.	[51]
SD rats	Controlled cortical impact injury	↑ Free choline in surrounding of injured area after 24 h of injury	Scremin et al.	[45]
C57BL6 mice	Controlled cortical impact injury	↓ cortical and cerebellar PC and SM ↑ hippocampal PC and SM after 3 months of injury	Abdullah et al.	[47]
C57BL6 mice	Closed head injury	↓ plasma PC and lyso-PC after 3, 12 and 24 months of injury	Emmerich et al.	[49]
C57BL6 mice	Closed head injury	↑ cortical and hippocampal PC, lyso-PC and SM after 24 h, and 3, 6, 9 and 12 months of injury	Ojo et al.	[48]
C57BL6 mice	Controlled cortical impact injury	↑ SM in brains after 2 and 7 days of injury	Novgorodov et al.	[52]
C57BL6 mice	Controlled cortical impact injury	↑ Lyso-PC in lysosomal membranes of injured cortices after 1 h f injury	Sarkar et al.	[53]
Sabra rats	Weight drop method	75, 81, and 245% ↑ PLA_2_ activity after 15 min, 4 and 24 h of injury resulted in respective elevation of fatty acid release after aminocaproylphosphatidylcholine catalysis	Shohami et al.	[54]
Rats	Controlled cortical impact injury	↑ PC in mid brain and thalamus after 14 days of injury	Li et al.	[55]
SD rats	Controlled cortical impact injury	↑ PC and lyso-PC in white and grey matter after 1 and 3 h of injury	McDonald et al.	[56]
**Clinical Studies**				
**Patients**	**Brain Injury**	**Observations**	**Authors**	**Ref.**
10	Fall/vehicle crash	Highest lyso-PC on day 1 and highest PC on day 4 was detected in CSF	Pasvogel et al.	[50]
40	Vehicle accidents	↑ regional choline/creatinine ratio estimated during 1–16 days after injury	Holshouser et al.	[57]
26	Accidental head injuries	↑ choline/creatinine and ↓ NAA/choline ratios in white matter during 3–38 (mean 11 days) days after injury	Garnett et al.	[58]
25	Mild head injuries	↑ NAA/choline ratio capsula interna and cerebral peduncles estimated during 1–20 days after injury	Kubas et al.	[59]
45	Fall/vehicle accidents	↑ choline/creatinine and ↓ NAA/choline ratios during 6–12 months after injury	Holshouser et al.	[60]
42	Severe brain injuries	↑ choline levels in occipital gray matter and parietal white matter after initial 7 days of injury	Eisele et al.	[61]
NA	Vehicle accidents	Highest PC within 24 h was found in CSF	Parsons et al.	[62]
10	Fall/vehicle accidents	↑ choline ratios in central brain after 48–72 h of injury	Marino et al.	[63]
8	Severe brain injuries	↑ choline/creatinine and ↓ NAA/choline ratios in occipital gray matter and parietal white matter after 5 months of injury	Yoon et al.	[64]

PCh (phosphocholine), GPC (glycerophosphocholine), PC (phosphatidylcholine), SM (sphingomyelin), lyso-PC (lysophosphatidylcholine), NAA (n-acetyl aspartate), ↑ (increased), ↓ (decreased).

### 5.1. Post-TBI Choline Changes during Subacute, Acute and Chronic Phases Evident from Neuroimaging

**Figure 5:**
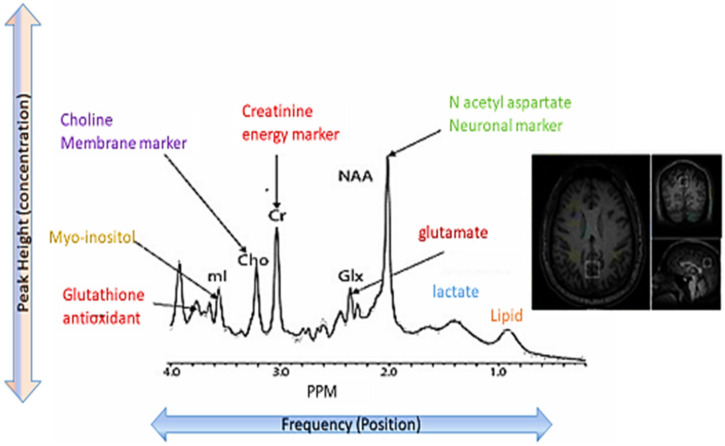
Magnetic resonance spectroscopy measuring major metabolites and providing a window into primary pathophysiological changes happening after TBI. The spectrum denotes the points mI, Cho, Cr, Glx and NAA representing myoinositol, choline, creatinine, glutamate and N-acetyl aspartate, respectively. In detail, myoinositol is a glial marker while choline is a membrane marker. Creatine is linked to mitochondrial function and glutamate is an excitatory neurotransmitter. The biggest spike of N-acetyl aspartate on the spectrum is related to the number of working neurons. (Adopted and modified from [77]).

Neuroimaging techniques are useful for the evaluation and prognostication of TBI patients. Magnetic resonance spectroscopy (MRS) is a non-invasive technique that allows the quantification of metabolites in brain tissues on the basis of resonance frequencies and is employed to assess pathological metabolic abnormalities [60] (Figure 5). The phospholipids exist in the entire brain but normally are not visible through MRS. However, under certain pathological conditions, i.e., TBI, they are degraded, liberated and become detectable [65]. Choline is the marker used to assess the damage to brain cells resulting in membrane breakage. Normally, the brain has 0.5–2.5 mmol/L choline [66] that tends to increase after pathological changes in the membranes [57]. This choline peak obtained through the proton MRS is centered at 3.2 ppm and is constituted by free choline, phosphocholine and glycerophosphocholine, which are involved in the metabolism of phosphatidylcholine. [67].

The increase in choline is considered as a marker of post-TBI membrane disruptions, which gives an insight into the pathological changes happening during the initial days after injury. Mostly, the choline-comprising phospholipids are not soluble under normal physiological conditions. The TBI-induced membrane turnover causes an increase in choline levels, which become visible by magnetic resonance spectroscopy (MRS) [68]. Many researchers have attempted to find the post-TBI chemical changes in the brains of individuals during acute time frames. Proton magnetic resonance spectroscopic imaging (1 H-MRSI) of ten patients was carried out by Marino et al. during subacute and acute phases of brain trauma; increased choline/total metabolites were reported in 5/10 patients [63]. TBI-induced diffuse axonal injury and altered metabolite ratios were estimated by Holshouser et al. in 40 children during the acute time frame after injury. Significantly increased choline levels were noted by MRS in hemorrhagic brains as compared to the healthy control [57]. Ashwal et al. studied 26 infants and 27 children with TBI and reported the elevated choline/creatinine ratios in the acute phase [69]. Shutter et al. also found elevated choline levels through MRS of forty-two severely injured patients after seven days of brain trauma [70]. During the acute phase of TBI, the levels of choline-comprising metabolites tend to increase, due to shearing damage to the cellular membrane. These findings were in line with Eisele et al., who reported that the choline peak on MRS is correlated with the post-TBI myelin breakdown [61].

In a study by Garnett et al., twenty-six TBI patients were examined through MRI/MRS in acute and chronic phases scheduled at mean 12 days and 6.2 months, respectively. In comparison to healthy individuals, increased choline/creatine levels were noted both acutely and chronically in these TBI patients [58]. Delayed choline quantification was done after 5 months of TBI in another study involving 8 TBI patients. In comparison to healthy controls, 1H-MRS investigation demonstrated choline/creatine in comparison to 14 healthy controls [64]. Friedman et al. also reported the increased choline levels in the occipital grey matter during chronic stages of TBI by providing the MRS evidence of cellular injury [71]. During the chronic phase, the increased choline might be due to diffuse glial proliferation that is corroborated by enhanced levels of myoinositol, which persists for months after injury [72]. Another explanation of this elevated choline in the chronic phase of TBI is the hyperosmolarity state of white matter leading to the detection of increased choline [68,73].

**Figure 6:**
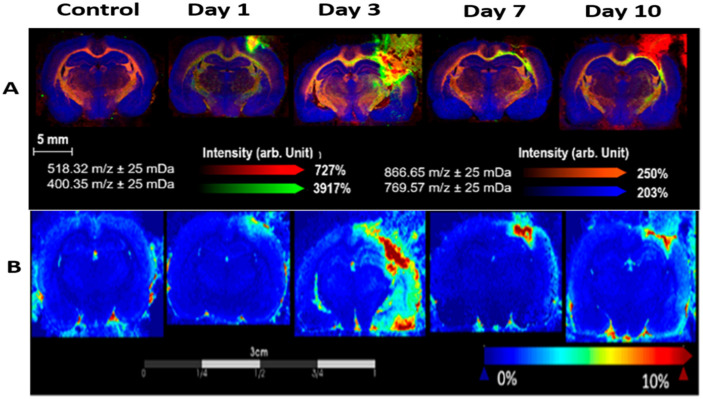
The post-TBI altered lipidomic profile revealed by MALDI-MSI shows the expression of (**A**) palmitoylcarnilite and (**B**) lyso-phosphatidylcholine in caudal sections of brain at different time points after brain injury. This figure was adopted and modified from Mallah et al. [75].

The role of lipid homeostasis is known to have a correlation with the severity of brain trauma. Imaging mass spectrometry is also used to visualize the lipid dynamics and molecular changes occurring in the injured brain. In a study by Mallah et al., lipid changes were tracked by MALDI-MSI (matrix-assisted laser desorption/ionization mass spectrometry imaging) in rats exposed to controlled cortical brain injury and identified the lipid alterations occurring at injury sites and distant regions [74]. To understand the role of lipid changes in the post-TBI inflammation and regeneration process, another study by Mallah et al. identified the new lipid markers called acylcarnitines at different time points after injury. The expression of acylcarnitine was found at its maximum in the acute phase of injury, as shown in Figure 6 [75]. Guo et al. also reported that the levels of docosahexaenoic acid are prominently elevated during the acute phase of injury [76].

### 5.2. Post-TBI Alternation in the Central Cholinergic System

Acetylcholine is one of the important neurotransmitters involved in maintaining neuronal plasticity and cognition. Structurally, it comprises the choline molecule esterified with acetic acid. The post-TBI damages to the central cholinergic system persist from days to months and the preservation of this deteriorating cholinergic functionality in the acute phase of injury might be a potential therapeutic strategy [78].

The post-TBI cholinergic dysregulation plays as one of the key contributors to acute and chronic neuropathology. After brain trauma, the levels of acetylcholine are massively increased in the acute phase, as evident by the exaggerated cholinergic levels in human cerebrospinal fluid, which also causes the precipitation of epilepsy [79]. The reduction in muscarinic acetylcholine receptors has been observed in rats and newborn piglets at 24 h and 6 h of brain injury, respectively. Additionally, the binding of α7- nicotinic acetylcholine receptors was also noted to decrease in various brain regions of rats subjected to brain trauma during acute as well as chronic phases of TBI [80].

Choline acetyltransferase (ChAT) is an enzyme present presynaptically and involved in the synthesis of Ach. There is sufficient preclinical and clinical evidence revealing the post-TBI downregulation of ChAT contributing towards the loss of cholinergic neurons and reduced ChAT protein [81,82,83]. The cholinergic neurotransmission is also regulated by the vesicular ACh transporter (vChAT), which is a well-known enzyme that transports ACh into vesicles. The enzyme is downregulated in the acute phase of TBI in multiple regions of the brain, as evident by preclinical models of moderate TBI [84,85]. However, its upregulation takes place in chronic periods due to compensatory mechanisms, which result in behavioral improvements [86]. Furthermore, the activity of acetylcholinesterase (AChE) is also increased in the acute phase of TBI and this upregulation might be a compensatory response to regulate the elevated Ach levels after TBI [87].

Like various neurodegenerative disarrays, post-TBI neuropsychiatric deficits result from disrupted homeostatic mechanisms, eventually leading to deteriorated molecular machinery and ineffective neurotransmission [79]. During chronic periods of TBI, the cholinergic neurotransmission keeps on changing and exerts an impact on long-term post-TBI behavioral responses. Many animal and autopsy studies highlight the increased susceptibility of cholinergic neuronal damage in the forebrain, resulting in increased vulnerability of senile plaques and tau protein deposition, and contributive to compromised cholinergic neurotransmission in chronic TBI [79].

During chronic phases of TBI, hypo-functionality of the cholinergic system is also precipitated by decreased ACh synthesis, release and altered acetylcholinesterase activity. The TBI-induced degeneration of α7- nicotinic acetylcholine receptors occurs due to cholinergic excitotoxicity, resulting in further deterioration of cholinergic neuronal circuitry [78].

## 6. TBI-Associated Neurological Comorbidities

The consequences of chronic TBI put the survivors at a huge risk of developing several disorders, as brain trauma initiates a series of immediate or delayed pathological events. The disruption of the blood-brain barrier and neuroinflammatory processes collectively result in the exacerbation of long-term complications as an alteration in the array of cellular events; this results in neurodegeneration, neuronal loss, synaptic variations and brain atrophy [88]. The dysregulated neurotransmitters in TBI also exert crucial impact on domains involved with behavioral homeostasis and resulting in neurobehavioral sequelae [89]. The correspondence between choline changes and post-TBI neurological disorders are hereby reviewed.

### 6.1. Alzheimer’s Disease (AD)

Alzheimer’s disease is a progressively developing neurodegenerative disorder involving the extracellular deposition of diffused neuritic plaques comprising amyloid beta peptide and intracellular neurofibrillary tangles of tau proteins. The amyloid precursor protein (APP) has a key role in the progression of AD, as this protein undergoes the sequential proteolytic cleavages to yield β-amyloid peptides (Aβ) [90]. The literature reveals the existence of the epidemiological relationship between the development of AD and TBI, as TBI is the strongest non-genetic risk factor for AD [91]. A TBI-induced cognitive deficit is directly proportional to the severity of brain injury. The location of temporal lobes in the skull makes them vulnerable to trauma and any resulting damage to the hippocampus plays a vital role in post-TBI cognitive impairment [92]. During Alzheimer’s disease, amyloid peptide (Aβ4) promotes the degradation of phosphatidylcholine and causes the leakage of choline and activation of PLA2. Glycerophosphocholine (GPCh) is produced from phosphatidylcholine, which further causes the aggregation of Aß4 and also catabolize to give choline [39].

Mulder et al. noticed the altered metabolism of choline-comprising phospholipids in AD brains, as the lyso-PC/PC ratio was reduced in CSF of AD patients [93]. In a 5-year observational study by Mapstone et al., the cognitively normal older adults had depleted phosphatidylcholine metabolites in their plasma and were predicted to phenoconvert to AD within 2–3 years [94]. The diminished levels of three phosphatidylcholines (16:0/20:5, 16:0/22:6 and 18:0/22:6) were also reported by Whiley et al. in the plasma of AD patients [95]. The postmortem brain examination showed the pathological parallels between TBI and AD [96]. Brain trauma causes the upregulation of amyloid precursor protein (APP), resulting in the accumulation of APP in injured axons, which is cleaved abnormally to the amyloid-beta (Aβ) protein [97]. TBI also deregulates the apolipoprotein E, which also influences the amyloid pathology [98]. The association of the APOε4 genotype with elevated Aβ deposition is another risk for developing late-onset AD following TBI [98].

The Gaudin et al. observed that the phosphatidylcholine dysregulation is crucial in AD, as phospholipases (PLA2 and PLD) are linked to Aβ activation [99]. Overactivated PLA2 after TBI causes the accelerated breakdown of membrane phospholipids and a strong correlation exists between PLA2 activation and the progression of AD. Furthermore, the PLA2-mediated conversion of phosphocholine into glycerophosphocholine and the loss of choline take place, resulting in the degradation of cholinergic neurons [39]. Due to the association between cholinergic neurotransmission and cognitive processes, the loss of cholinergic functions is believed to be an important contributor to cognitive impairment, which is a shared pathophysiological characteristic of both AD and TBI [100]. Furthermore, the overactivation of PLD after TBI causes the catalysis of phosphatidylcholine to phosphatide and is directly related to AD, as unusual phosphatidic acid signaling is linked to neurodegenerative processes.

### 6.2. Parkinson’s Disease

Brain trauma synergistically accelerates the pathophysiology of Parkinson’s disease (PD), which is a neurodegenerative condition developed due to the loss of dopaminergic neurons in the substantia nigra. The recent findings suggest that the risk of PD is 56% in patients suffering from mild TBI but the danger is raised to 83% when the severity of TBI changes from moderate-severe. Gardner et al. revealed that within 12 years, 949 out of 1462 veterans developing PD previously had certain brain trauma [101]. Thus, TBI is known to be the chief epigenetic risk factor for Parkinson’s disease, as few neurons become more vulnerable to PD pathology after diffused axonal damage happens during TBI [102].

Phosphatidylcholines give structural integrity to membranes as well as influence the cell signaling and activation of several enzymes. Due to TBI, the rapid hydrolysis or enzymatic degradation of phosphatidylcholines causes the generation of lysophosphatidylcholine. This lyso-PC has the role in the activation of pro-apoptotic Bid protein and caspase-3. Bid belongs to the Bcl-2 family, which is involved in cellular destructive processes, mitochondrial dysfunction and TNF- α mediated apoptosis [103]. Furthermore, the caspase-3 activation is considered a hallmark in PD, as it might promote neuronal apoptosis and microglial activation [104]. The lysoPC is also reported to reduce the expression of the anti-apoptotic factor called the TNF receptor-associated factor (TRAF) [103]. Altogether, these actions exerted by increased PC and lysoPC promote apoptosis, which is one of the crucial factors responsible for the precipitation of dopaminergic neuronal death in the brain.

Hartmann et al. describe that neuroinflammation mediated by TNF works fundamentally in the pathogenesis of PD, with increased TNF levels detected in the CSF and postmortem brains of patients with PD [105]. LysoPC species cause chemotaxis of macrophages and T-lymphocytes to injured brain tissue and also play a role in the release of various inflammatory mediators, including TNF-α [106].

### 6.3. Epilepsy

Recurrent seizures as a comorbidity of TBI are becoming a universal challenge for brain health due to the increasing incidence of brain trauma. A total of 20% of the general population develop symptomatic post-traumatic epilepsy (PTE) within 1–2 years of injury but this incidence increases up to 50% in military personnel [107]. The pathophysiology of TBI-induced epileptogenesis includes increased excitotoxicity and free radical generation, due to accumulated glutamate and iron deposition, respectively [108,109].

The TBI-induced cerebral ischemia causes excessive glutamate release, which elevates intracellular Ca^2+^ levels leading to excitotoxicity and precipitation of seizures. The cerebral ischemia also causes the activation of phospholipases, including PLA2. Thus, increased phosphatidylcholine metabolism results in the activation of the inflammatory cascade. This post-TBI neuroinflammation can exist for months and contribute towards the precipitation of PTE. The literature reports the increased catabolism of phosphatidylcholine during both seizures and TBI, which elevates free choline and free fatty acids [110]. Imran et al. reported in a real-time microdialysis study that phospholipid hydrolysis is accelerated in hyperactive neurons during seizures, resulting in a two-fold increase in extracellular choline levels [27,111]. Furthermore, the levels of 8-Isoprostanes, which is an in vivo indicator of oxidative stress and membrane breakdown, were significantly increased (3–4 folds) during the time-course of status epilepticus in lithium–pilocarpine-induced acute status epileptic rats [27]. The postmortem increase in levels of free choline is also documented to confirm the enhanced hydrolysis of phosphatidylcholine in the convulsive brain [112].

### 6.4. Depression

Due to the struggle with a momentary or lifelong disability, depression is another psychiatric complication reported in post-TBI survivors. As the TBI results in damage to different brain areas, the injury to the part of the brain controlling emotions might result in altered neurochemical levels and precipitate mood changes [113]. A longitudinal study concerning TBI participants reported a 31% incidence of moderate-severe depression at 1 month after injury [114]. Jorge et al. revealed the increased lifetime prevalence in patients sustaining head injuries [113].

The study on post-mortem brains revealed the overexpression of PLA2 in the cortex of depressed patients [115]. Phosphatidylcholines in cell membranes are targeted by this overexpressed PLA2, leading to lysophospholipids and arachidonic acid generation, which further participate in the generation of numerous inflammatory mediators [116]. Lithium, which is commonly employed in bipolar and unipolar depressive disorder, works through the inhibition of the overactivated PLA2 in the brain [117]. During TBI, the activity of sphingomyelinase is also increased, resulting in increased phosphocholine and ceramide in the brain. Antidepressants are also supposed to reduce this enzymatic activity in a dose-dependent style [118].

## 7. Choline-Specific Therapeutic Strategies for the Amelioration of TBI and Coexisting Neurological Diseases

Choline plays a key role in the biosynthesis of Ach and various phospholipids, i.e., phosphatidylcholine, lyso-PC, sphingomyelin and choline plasmalogen. Its role in neurogenesis and memory development is well established and its deficiency might lead to neural tube defects [119]. Lecithin is an easily available nutraceutical and works as a precursor of choline. Lecithin levels are directly correlated with the levels of choline and acetylcholine, as it stimulates the Ach synthesis in the brain, due to increased levels of choline [120]. A comparative study demonstrated that plasma choline levels were increased up to 400% with supplements of exogenous purified lecithin, as compared to a diet with low choline content [121]. A study conducted on rats fed with lecithin derived from soybeans or eggs found that forms of lecithin increased brain choline, blood choline and Ach synthesis in the brain [122]. Lecithin, alone and in combination, has been tested for the potential to regress the progression of dementia and AD in 21 studies and 12 randomized trials [35]. Clear evidence supporting its role in the amelioration of dementia and AD is not established.

CDP-choline or 5’-cytidinediphosphocholine, generically known as citicoline has been employed clinically in the management of TBI [123]. In TBI, lipid peroxidation by phospholipases [124], i.e., PLA2, has been considered to play a crucial role in the pathophysiology of TBI; the number of experimental studies demonstrated that the PLA2 inhibition by CDP-choline exhibited a beneficial effect in brain injury [125]. During in vivo studies, it corrected the BBB dysfunction and combated the edema and neuronal death in an experimental model of TBI [126].

**Figure 7:**
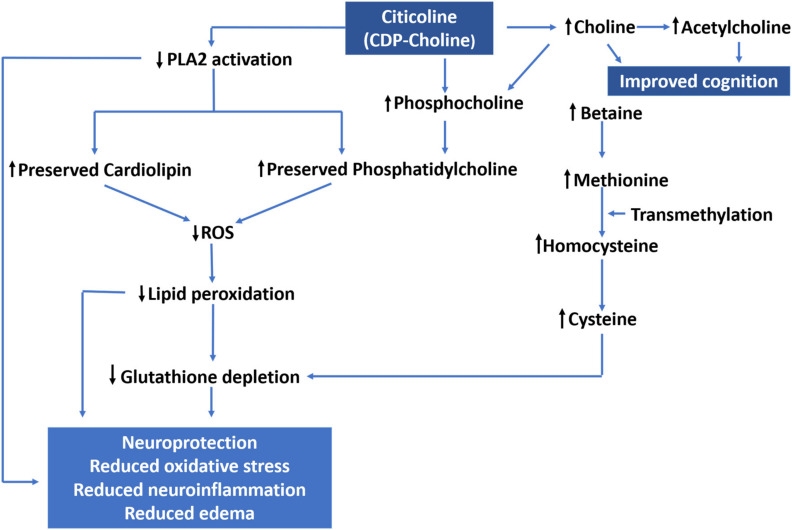
Proposed mechanism of action of Citicoline (CDP-choline) to ameliorate the pathogenesis of TBI. Citicoline decreases the expression of PLA2, resulting in the preservation of cardiolipin and phosphatidylcholine in the brain, which eventually result into reduced (↓) oxygen species andlipid peroxidation and increased (↑) glutathione levels, which is lsimultaneously supplemented through the cysteine–choline pathway as well. On the other hand, the citicoline also increases acetylcholine, boosts cholinergic neurotransmission and post-TBI cognition. Phosphocholine generated from citicoline also directly yields phosphatidylcholine, the essential constituent of the membrane phospholipid.

After administration, it quickly yielded choline, which is used to synthesize phosphatidylcholine through the CDP-choline pathway. Hence, it provides neuroprotection by improving the synthesis of phosphatidylcholine. Furthermore, it has the capacity to combat oxidative stress via improving glutathione levels [127]. CDP-choline works as a donor of choline to take part in the synthesis of Ach as shown in Figure 7. A study on rats evaluated the impact of CDP-choline on the post-TBI neurobehavioral deficit. The treatment with CDP-choline resulted in the attenuation of cognitive deficit of animals by increasing the levels of ACh in the hippocampus and cortex [128]. In clinical trials, CDP-choline is found safe when administered at 2 g/day, thus it might be employed as a part of combination therapy in TBI [4]. Misbach et al. presented the outcomes of the first double-blinded and placebo-controlled clinical trial in which authors reported the association of citicoline with rapid recovery of TBI [129]. The CDP-choline was clinically evaluated by Richer and Cohadon in 60 patients with acute head trauma. The intravenous administration of 750 mg/day of CDP-choline resulted in improved consciousness [130]. In another study by Lozano, the 78 patients with cranio-encephalic trauma had prominently reduced cerebral edema and faster recoveries, leading to shorter hospital stays [131]. Another randomized double-blinded clinical trial was conducted by Shokouhi et al., in which 58 TBI patients treated with citicoline resulted in protection against inflammatory damage in TBI patients [132].

Alpha-glyceryl phosphorylcholine (α-GPC) is semi-synthetically derived from lecithin. After administration, it is converted into the metabolic active form of choline, phosphorylcholine. Phosphoryl choline reaches the cholinergic nerve terminals and stimulates Ach synthesis [35]. α-GPC has shown improved cognitive health by increasing the hippocampal Ach levels; its efficacy in ameliorating dementia and AD is proved [133]. It is also reported to show cognitive improvement by increasing neuroblast formation, reducing neuronal death and BBB disruption in animals suffering from seizures, suggesting its significance in improving cognition in epileptic patients [134].

Fortasyn connect (FC) is a multi-nutrient combination comprising choline uridine, a cofactor needed for phospholipid synthesis, vitamins and polyunsaturated omega- 3 fatty acids. The one-week administration of FC to mice with controlled cortical impact injury showed improved cognition and remyelination. The enhanced phospholipid biosynthesis promoted by FC supplementation also resulted in reduced contusive lesion size, which might be the reason behind improved cognitive outcomes [135]. A double-blinded placebo-controlled trial of Fortasyn was carried out in a group of 311 patients with prodromal Alzheimer’s disease. The outcomes of the study showed that once-daily oral administration of this multi-nutrient worked as a source of brain phospholipid precursors, which rescued the hippocampal atrophy and slowed down the cognitive impairment. [136]. Overall, FC is reported to provide these beneficial effects by regulating neurogenesis, synaptic plasticity and neural circuitry [135,137].

The anti-cholinesterases, i.e., physostigmine and donepezil, have also been employed in the management of cognitive impairment faced by TBI patients. The beneficial effects were observed in two case studies, where patients with severe TBI received physostigmine, which ameliorated the disorientation and memory loss [138,139]. Similarly, donepezil given to two patients with TBI resulted in better alertness and memory reconciliation [140]. In an open-label trial by Whelan et al., 53 TBI patients were treated with donepezil and improved neuropsychiatric outcomes were yielded [141]. Various preclinical and clinical studies showing the benefits of choline-targeted therapies in improving the post-TBI neurological function are shown in Table 4.

## 8. Conclusions

Depending on the degree, TBI causes direct structural damage in the brain resulting in a range of acute and chronic pathophysiological consequences precipitating the altered neurological functions. The TBI-induced overactivated phospholipases cause the disruption of phospholipids and changes in levels of choline and associated phospholipids, which work as an initial hallmark and are directly correlated with the severity of brain damage, in addition to possibly persisting over time. The altered choline leads to insufficient cholinergic neurotransmission and impaired neurogenesis, resulting in comorbid neurodegenerative manifestations. The possible outcomes of CDP-choline supplementation in the management of TBI and associated neurobehavioral conditions were mostly investigated in preclinical and clinical studies. On the basis of the literature, the post-TBI choline changes with its contribution to various cellular pathologies are obvious and its correction might be one of the approaches to treat those affected by TBI.

## Figures and Tables

**Table 1:** The definitive details of calculation of Glasgow coma scale score and description of Glasgow coma scale (GCS) and post-traumatic amnesia (PTA) classification systems employed to categorize traumatic brain injury.

Glasgow Coma Scale Score Calculation					
Eye Opening Response	Score	Verbal Response	Score	Motor Response	Score
Spontaneous	4	Oriented	5	Obeys commands	6
Response to verbal command	3	Confused	4	Localizing response to pain	5
Response to pain	2	Inappropriate words	3	Withdrawal response to pain	4
No eye-opening	1	Incomprehensible speech	2	Flexion to pain	3
		No verbal response	1	Extension to pain	2
				No motor response	1

**Table 2:** Defining the severity of traumatic brain injury on the basis of Glasgow coma scale (GCS) and post-traumatic amnesia (PTA) classification systems.

Severity of Traumatic Brain Injury			
Classification System	Mild	Moderate	Severe
GCS scale	13–15	9–12	3–8
PTA scale	Less than 1 day	From 2 to 7 days	More than 7 days

**Table 4:** Preclinical and clinical studies reporting the improvement of brain function through providing the choline-targeted post-TBI therapies.

**Pre-Clinical Studies**					
**Animal**	**TBI Model**	**Treatment and** **Schedule**	**Observation**	**Author**	**Ref.**
SD rats	Controlled cortical impact	Dietary choline supplementation for 2 weeks	Improved memory and reduced neuroinflammation	Guseva et al.	[142]
SD rats	Controlled cortical impact	CDP-Choline 100 mg/kg i.p. for 18 days	Increase Ach release and decreased spatial memory deficit.	Dixon et al.	[128]
SD rats	Controlled cortical impact injury	CDP-Choline 100, 200 and 400 mg/kg given i.p. immediately and 6 h after TBI	Decrease neuronal loss and contusion volume with improved neurologic recovery	Dempsey et al.	[126]
SD rats	Controlled cortical impact	CDP-Choline 100 and 400 mg/kg i.p. given twice after TBI	Reduced edema in injury area with decreased BBB breakdown	Baskaya et al.	[143]
Wistar rats	Blunt Trauma	Citicoline 250 mg/kg i.p.	Reduced oxidative stress	Menku et al.	[144]
SD rats	Closed head injury	Citicoline 250 mg/kg injected i.v. 30 min and 4 h after injury	Decreased brain edema, BBB permeability, axonal and myelin sheath damage and reduced oxidative stress.	Qian et al.	[145]
SD rats	Controlled cortical impact injury	Citicoline 200 mg/kg i.p. Started 4 h after surgery and continued until five injections.	Reduced post-TBI cognitive impairment	Jacotte–Simancas et al.	[125]
Wistar rats	Chronic hypoperfusion	Citicoline 500 mg/kg i.p. for 21 days	Prevented white matter damage and enhanced cognition	Lee et al.	[146]
C57BL/6 mice	Controlled cortical impact injury	Fortasyn added to diet for 70 days	Improved cognition and neurogenesis with less oligodendrocyte loss	Thau–Zuchman et al.	[135]
**Clinical Studies**					
**Patients**	**Study Design**	**Treatment**	**Treatment Schedule**	**Author**	**Ref.**
216	Single-blinded randomized study	CDP-choline 4 g/day divided in 4 doses give i.v. on day 1–2 followed by 3 g/day divided in three doses for days 3–4 and continued as 200 mg orally every 8 h after discharge from ICU	Overall improvement in patient’s status, reduced physical dependency and better social reinsertion	Maldonado et al.	[147]
272	Double-blinded placebo-controlled study	CDP-choline 1000 mg CDP-choline i.v. daily for 14 days	Improved consciousness of patients as compared to placebo	Tazaki et al.	[148]
10	Placebo-controlled study design	CDP-choline 1 g/d p.o. for 3 months	Normalization of cerebral blood flow and enhanced memory	Carri ’on et al.	[149]
14	Double-blinded placebo-controlled study	CDP-choline 1 g p.o. for 1 month	Improved cognition as compared to placebo	Levin et al.	[150]
28	Placebo-controlled randomized trial	Citicoline 1 g i.v, for 14 days	Improved neuroprotection yielded in patients	Lazowsk et al.	[151]
2706	Systematic review and meta-analysis	Citicoline 250 mg to 6 g per day, administered orally or parenterally for 7–90 days	Beneficial health outcomes and with no safety concerns	Secades et al.	[152]
134	Retrospective matched pair analysis	Citicoline 3 g/day by i.v. for 21 days	The early administration of citicoline resulted in better outcomes	Trimmel et al.	[153]
40	Double-blinded randomized clinical trial	Citicoline 500 mg/6 h or 2 g/day i.v. for 15 days	Treatment of patients resulted in reduced MDA levels	Salehpour et al.	[154]
16	Double-blinded placebo-controlled study	Lecithin 16 g/day divided in two doses was given for 30 days	Improved cognition was observed	Levin et al.	[155]

## Data Availability

The data presented in this study are available on request from the corresponding authors.
